# TENAYA and LUCERNE: Rationale and Design for the Phase 3 Clinical Trials of Faricimab for Neovascular Age-Related Macular Degeneration

**DOI:** 10.1016/j.xops.2021.100076

**Published:** 2021-11-17

**Authors:** Arshad M. Khanani, Robyn H. Guymer, Karen Basu, Heather Boston, Jeffrey S. Heier, Jean-François Korobelnik, Aachal Kotecha, Hugh Lin, David Silverman, Balakumar Swaminathan, Jeffrey R. Willis, Young Hee Yoon, Carlos Quezada-Ruiz

**Affiliations:** 1 Sierra Eye Associates, Reno, Nevada; 2 Reno School of Medicine, University of Nevada, Reno, Nevada; 3 Centre for Eye Research Australia, Royal Victorian Eye and Ear Hospital, University of Melbourne, Melbourne, Australia; 4 Roche Products (Ireland) Limited, Dublin, Ireland; 5 Roche Products Ltd., Welwyn Garden City, United Kingdom; 6 Ophthalmic Consultants of Boston, Boston, Massachusetts; 7 Ophthalmology Department, Centre Hospitalier Universitaire Bordeaux, Bordeaux, France; 8 University of Bordeaux, INSERM, BPH, U1219, Bordeaux, France; 9 Genentech, Inc., South San Francisco, California; 10 F. Hoffmann-La Roche Ltd., Mississauga, Ontario, Canada; 11 Asan Medical Center, University of Ulsan, Seoul, South Korea; 12 Clinica de Ojos Garza Viejo, San Pedro Garza Garcia, Nuevo Leon, Mexico

**Keywords:** Anti-VEGF therapy, Faricimab, Neovascular age-related macular degeneration, Personalized treatment interval, BCVA, best-corrected visual acuity, CNV, choroidal neovascularization, CRC, central reading center, CST, central subfield thickness, FFA, fundus fluorescein angiography, nAMD, neovascular age-related macular degeneration, PTI, personalized treatment interval, T&E, treat-and-extend, VEGF, vascular endothelial growth factor

## Abstract

**Purpose:**

To describe the design and rationale of the phase 3 TENAYA (ClinicalTrials.gov identifier, NCT03823287) and LUCERNE (ClinicalTrials.gov identifier, NCT03823300) trials that aimed to assess efficacy, safety, and durability of faricimab, the first bispecific antibody for intraocular use, which independently binds and neutralizes both angiopoietin-2 and vascular endothelial growth factor-A (VEGF-A) versus aflibercept in patients with neovascular age-related macular degeneration (nAMD).

**Design:**

Identical, global, double-masked, randomized, controlled, phase 3 clinical trials.

**Participants:**

Adults with treatment-naïve nAMD.

**Methods:**

These trials were designed to evaluate patients randomized to receive faricimab 6.0 mg up to every 16 weeks after 4 initial every-4-week doses or aflibercept 2.0 mg every 8 weeks after 3 initial every-4-week doses. The initial doses in the faricimab arm were followed by individualized fixed treatment intervals up to week 60, based on disease activity assessment at weeks 20 and 24, guided by central subfield thickness, best-corrected visual acuity (BCVA), and investigator assessment. The primary efficacy end point was BCVA change from baseline averaged over weeks 40, 44, and 48. Secondary end points included the proportion of patients receiving every-8-week, every-12-week, and every-16-week faricimab and anatomic outcomes. Safety outcomes included incidence and severity of ocular and nonocular adverse events. From week 60, faricimab-treated patients followed a personalized treatment interval (PTI), a novel protocol-driven treat-and-extend regimen with interval adjustment from every 8 weeks to every 16 weeks based on individualized treatment response measured by anatomic criteria, functional criteria, and investigator assessment of patients’ disease activity.

**Main Outcome Measures:**

Rationale for trial design and PTI approach.

**Results:**

The TENAYA and LUCERNE trials were the first registrational trials in nAMD to test fixed dosing regimens up to every 16 weeks based on patients' disease activity in year 1 and incorporate a PTI paradigm during year 2. The PTI approach was designed to tailor treatment intervals to individual patient needs, to reflect clinical practice treatment practice, and to reduce treatment burden.

**Conclusions:**

The innovative trial design rationale for the TENAYA and LUCERNE trials included maximizing the benefits of angiopoietin-2 blockade through dosing up to every 16 weeks and PTI regimens based on patients' disease activity while fulfilling health authority requirements for potential registrational efforts.

Neovascular age-related macular degeneration (nAMD), characterized by abnormal growth of blood vessels into the macula, affecting the outer retina, and impacting photoreceptor integrity, is a leading cause of irreversible vision loss globally in adults 50 years of age and older if left untreated.^1^ Introduction of intravitreal anti–vascular endothelial growth factor (VEGF) therapy, including ranibizumab,^2^^,^^3^ aflibercept,^4^ bevacizumab (used off-label for ocular indications),^5^ conbercept,^6^ and, more recently, brolucizumab,^7^ administered at 4- to 12-week intervals, has improved vision outcomes dramatically and has reduced the risk of vision loss in patients with nAMD.^8^, ^9^, ^10^, ^11^

The efficacy and safety of intravitreal anti-VEGF therapy for nAMD are well established.^9^ However, best-achievable long-term outcomes require frequent injections and patient monitoring,^12^^,^^13^ a correlation further corroborated by data generated outside clinical trials.^14^, ^15^, ^16^, ^17^ Furthermore, replicating the schedule of visits and regimented treatment from clinical trials is difficult in clinical practice, often resulting in suboptimal dosing frequency correlated with loss of vision over time.^17^, ^18^, ^19^, ^20^ Even with optimal dosing frequencies, an observational study in a clincal setting reported that only approximately 20% of patients who received frequent intravitreal anti-VEGF injections during the first year of treatment were able to preserve their reading and driving vision until their death.^15^ In studies that extended to fixed every-12-week dosing regimens, intravitreal anti-VEGF monotherapy with ranibizumab failed to sustain the initial best-corrected visual acuity (BCVA) gains achieved during the loading phase in the first year of treatment. This was demonstrated in the EXCITE trial, in which only approximately 40% of patients treated with every-12-week ranibizumab were able to maintain initial BCVA gains,^21^ and in the PIER trial, in which the BCVA gains declined rapidly after the switch from monthly to quarterly ranibizumab dosing at month 3 and BCVA gains at year 2 were lower than those achieved with monthly dosing in previous trials of ranibizumab.^22^^,^^23^ An unmet need exists for treatments that achieve robust and sustainable BCVA gains over time with less frequent regimens than those currently available for intravitreal anti-VEGF monotherapy.

Because of the multifactorial nature of nAMD and despite tremendous achievement in treatment outcomes, underlying pathologic mechanisms associated with nAMD progression remain, such as vascular leakage and inflammation, that may not be addressed with intravitreal anti-VEGF monotherapy and that may lead to long-term detrimental effects on vision.^24^ This may explain the drop in BCVA beyond the first year of treatment with some intravitreal anti-VEGF monotherapy clinical trials.^12^^,^^25^ Thus, an unmet need exists for treatments that not only treat vascular leakage and neovascularization as with the current intravitreal anti-VEGF agents, but also address the concurrent inflammatory response leading to fibrosis and the ongoing cell death leading to atrophy.

Faricimab is a novel humanized bispecific immunoglobulin G1 monoclonal antibody designed for intraocular use that independently binds and neutralizes both angiopoietin-2 and VEGF-A.^26^^,^^27^ The efficacy and safety of faricimab were established in a phase 1 clinical trial, supporting further evaluation of faricimab.^28^ The phase 2 program for faricimab included the BOULEVARD trial in patients with diabetic macular edema and the AVENUE and STAIRWAY trials in patients with nAMD. The 36-week AVENUE trial established the efficacy and safety of faricimab compared with ranibizumab,^29^ and the 52-week STAIRWAY trial demonstrated sustained efficacy through extended durability of faricimab on fixed every-12-week and every-16-week dosing, with comparable vision and anatomic gains versus every-4-week ranibizumab.^30^

The phase 3 TENAYA (ClinicalTrials.gov identifier, NCT03823287) and LUCERNE (ClinicalTrials.gov identifier, NCT03823300) trials were designed to assess the efficacy, safety, and durability of faricimab, a bispecific antibody that targets both angiopoietin-2 and VEGF-A, building on the dosing regimen, design, and results from the phase 2 program in patients with nAMD.^29^^,^^30^ Herein we describe the rationale and methodology of the ongoing TENAYA and LUCERNE trials.

## TENAYA and LUCERNE Study Design and Rationale

### Study Overview

The TENAYA and LUCERNE trials are 2 identical, global, phase 3, multicenter, randomized, active comparator-controlled, double-masked, parallel-group, 112-week registrational studies, funded by F. Hoffmann-La Roche Ltd, to investigate the efficacy, safety, and durability of faricimab administered at up to 16-week intervals compared with on-label intravitreal aflibercept administered at 8-week intervals in treatment-naïve patients with nAMD. The trial design for faricimab in nAMD was a product of discussion with retina specialists around the world as well as input from global health authorities for registrational purposes, taking into account the different requirements for health authority approval of a new drug. The studies currently are ongoing and are being conducted in accordance with the International Conference on Harmonisation E6 guidelines for Good Clinical Practice and the principles of the Declaration of Helsinki, or the laws and regulations of the country in which the research is conducted. Written informed consent was obtained before initiation of any study procedures, and the study protocol was approved by institutional review boards (Table S1) before study start.

The trials enrolled 1329 patients (671 in TENAYA and 658 in LUCERNE) across 271 sites around the world. Patients were randomized in a 1:1 fashion to 2 treatment arms. Randomization was stratified by baseline BCVA Early Treatment Diabetic Retinopathy Study letter score as assessed on day 1 (≥ 74 letters, 73–55 letters, or ≤ 54 letters), low-luminance deficit on day 1 (< 33 letters or ≥ 33 letters), and region (United States and Canada, Asia, rest of the world). Low-luminance BCVA was measured by placing a 2.0-log unit neutral density filter (Kodak Wratten 2.0) over the best correction for that eye and having the participant read the normally illuminated Early Treatment Diabetic Retinopathy Study chart.

Patients randomized to the aflibercept arm received intravitreal aflibercept 2.0 mg every 4 weeks for 3 monthly initial doses and then continued on an every-8-week regimen up to week 108 according to the approved label of aflibercept. Patients randomized to the faricimab arm initially received intravitreal faricimab 6.0 mg every 4 weeks up to and at week 12 (4 injections) and then received every-16-week, every-12-week, or every-8-week dosing up to week 60 based on disease activity assessments at weeks 20 and 24. Disease activity assessments were guided by central subfield thickness (CST) and BCVA criteria and by investigator assessment. Patients with anatomic or functional signs of disease activity at weeks 20 or 24 were treated with every-8-week or every-12-week faricimab, respectively (Table 1). From week 28, patients receiving faricimab who did not have active disease at weeks 20 and 24 continued to be treated with faricimab at every-16-week intervals after already having completed 1 full every-16-week cycle after the last initiation dose at week 12.

**Table 1:** Disease Activity Criteria

Criterion	Disease Activity Criteria at Weeks 20 and 24
1	Increase of >50 μm in CST∗ compared with the average CST value over the previous 2 scheduled visits (weeks 12 and 16 for the week 20 assessment and weeks 16 and 20 for the week 24 assessment)
2	Increase of ≥75 μm in CST compared with the lowest CST value recorded at either of the previous 2 scheduled visits
3	Decrease of ≥5 letters in BCVA compared with average BCVA value over the previous 2 scheduled visits, owing to nAMD disease activity (as determined by the investigator)
4	Decrease of ≥10 letters in BCVA compared with the highest BCVA value recorded at either of the previous 2 scheduled visits, owing to nAMD disease activity (as determined by the investigator)
5	Presence of new macular hemorrhage (as determined by the investigator), owing to nAMD activity
6	Investigator opinion of significant nAMD disease activity at week 24 that requires immediate treatment (applies only at week 24)

BCVA = best-corrected visual acuity; CST = central subfield thickness; nAMD = neovascular age-related macular degeneration.

∗ Central subfield thickness to assess disease activity at weeks 20 and 24 was measured at the study site and was machine specific, whereas the CST value used in the personalized treatment interval phase is from the central reading center.

From weeks 60 to 108, patients randomized to faricimab received treatment according to a personalized treatment interval (PTI), further described below (in "Faricimab Dosing Regimens During the Second Year of the Study: Objective and Rationale for the Personalized Treatment Interval Regimen"). A study duration of 112 weeks allowed for evaluation of the role of faricimab in the treatment of patients with nAMD under a flexible, individualized regimen approach during the second year of the study.

These were double-masked studies, with the assessor physician, BCVA examiner, and patients masked to treatment assignment. All patients attended all visits, with a sham procedure administered when no study treatment was administered. No rescue treatments were permitted within the study, given that the purpose was to evaluate extended dosing with faricimab. However, patients could be withdrawn from the study if they were deemed to require rescue therapy.

### Study Participants and Eligibility Criteria

To participate in the trials, patients were required to have active treatment-naïve macular neovascularization (as described by the Consensus on nAMD Nomenclature Study Group,^31^ also called choroidal neovascularization [CNV], as referred to herein) secondary to nAMD and be 50 years of age or older on day 1. Patients were included if they were able to comply with study protocol and assessments in the investigators’ judgment. Additionally, patients had to have a BCVA between 78 and 24 letters (approximate Snellen equivalent, 20/32–20/320), assessed at the initial testing distance of 4 m on day 1.

Subfoveal, juxtafoveal, or extrafoveal CNV lesions were included as long as a subfoveal component related to CNV activity was identified on fundus fluorescein angiography (FFA) or OCT. Patients were included if they had a CNV lesion size of up to 9 disc areas on FFA with a CNV component area of more than 50% of the total lesion size on FFA.

Only 1 eye was assigned as the study eye. If both eyes were considered eligible (per the inclusion and exclusion criteria), the eye with the worse BCVA, as assessed at screening, was to be selected as the study eye.

Patients were excluded if, among other factors, they had CNV resulting from causes other than nAMD or had undergone cataract surgery or treatment for complications of cataract surgery with steroids or yttrium–aluminum–garnet laser capsulotomy within 3 months before day 1. A complete list of inclusion and exclusion criteria is provided in Table S2.

### Anatomic Assessments

Central reading centers (CRCs) conducted a masked assessment of patient eligibility during screening based on a review of color fundus photographs, OCT images, and FFA images to ensure that CNV secondary to AMD met the ocular inclusion criteria for the study. During the study treatment period, the CRCs provided a masked evaluation of all ocular images, including color fundus photographs, FFA images, optional indocyanine green angiography images, OCT images, and optional OCT angiography images to allow for accurate and unbiased assessment of the disease at study visits, including at weeks 20 and 24.

Because of the global nature of the trials executed across 271 sites, 2 CRCs, the Duke Reading Center and the Vienna Reading Center, needed to be included to manage the images and methods across a wide span of time zones and to provide site support and training, if needed. This highlighted the need to ensure that assessments could be reproduced and replicated by both CRCs. A harmonization process was undertaken that yielded excellent concordance in grading of images between the CRCs, and a manuscript on this exercise is in progress. For the trial, dual reads for CST were performed by 2 independent readers. If discrepancies were noted, these were adjudicated by a third reader. In the TENAYA and LUCERNE trials, spectral-domain OCT images were obtained with the Spectralis (Heidelberg Engineering, GmbH), Cirrus (Carl Zeiss Meditec), and Topcon (Topcon Medical Systems) devices.

### Rationale for Choice of Comparator and Comparator Dosing

Patients randomized to aflibercept were treated at every-8-week intervals starting at week 16 after the 3 every-4-week initiation doses (per its approved global posology) for the entire duration of the study. Although every-4-week ranibizumab was used as a comparator in the phase 2 program, aflibercept was chosen as a comparator in the faricimab phase 3 programs for both diabetic macular edema and nAMD because it has a single approved labeled dose (2.0 mg) and treatment regimen worldwide, which provides the same comparator in all countries for the phase 3 faricimab development program, satisfying regulatory requirements globally. Furthermore, aflibercept was dosed at fixed every-8-week intervals, providing a suitable comparator against which to evaluate the potential for extended dosing with faricimab dosed up to every 16 weeks. The decision to use aflibercept as a comparator aligned well with the findings from the 2020 American Society of Retina Specialists Preferences and Trends survey, which reported that aflibercept was the agent that retina specialists most commonly use as first-line therapy.^32^

### Faricimab Dosing Regimens During Year 1 of the Study

After the 4 monthly initiation doses of faricimab, patients subsequently were treated at fixed intervals ranging from every 8 weeks to every 16 weeks based on disease activity assessments at weeks 20 and 24. Disease activity criteria were prespecified and are noted in Table 1. Any of the disease activity criteria could be met to be deemed active. At week 20, 8 weeks after the last initiation dose, patients underwent the first disease activity assessment. If any of the criteria were met, they were treated with faricimab at that visit and then on a fixed every-8-week regimen until week 60.

**Figure 1:**
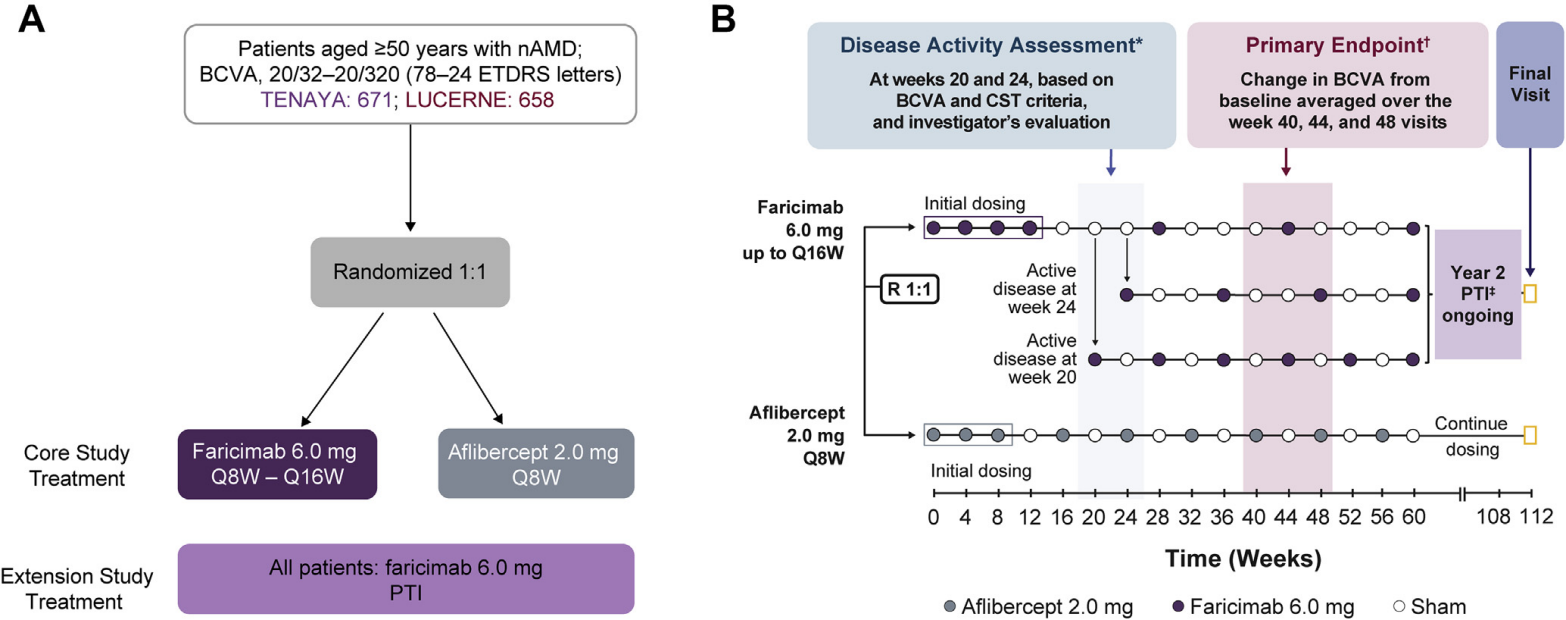
Diagram showing (A) study profile and (B) study design of the TENAYA and LUCERNE trials. ∗Protocol-defined assessment of disease activity at weeks 20 and 24. Patients with anatomic or functional signs of disease activity at these time points received treatment every 8 weeks (Q8W) or every 12 weeks, respectively. ^†^Change from baseline in best-corrected visual acuity (BCVA), as measured on the Early Treatment Diabetic Retinopathy Study (ETDRS) chart at a starting distance of 4 m, based on an average of the week 40, 44, and 48 visits. ^‡^Personalized treatment interval (PTI): interactive voice or web-based response systemguided flexible dosing in the faricimab arms starting at week 60. From week 60 onward, patients in the faricimab arm are treated according to a PTI dosing regimen between Q8W and every 16 weeks (Q16W). CST = central subfield thickness; nAMD = neovascular age-related macular degeneration; R = randomized.

At week 24, 12 weeks after the last initiation dose, patients underwent the second disease activity assessment, and if any criteria were met, they were treated and kept on a fixed every-12-week dosing regimen until week 60. Patients already receiving every-8-week treatment were assessed for disease activity at week 24, but were not treated because they already were receiving every-8-week treatment. Finally, the remaining patients who did not meet disease activity criteria at weeks 20 and 24 were treated at week 28 after the last initiation dose and continued on a fixed every-16-week regimen until week 60 (Fig 1).

### Rationale for Treatment Intervals

The treatment intervals in the phase 3 trials were based on the phase 2 STAIRWAY and AVENUE trial designs. The extended dosing was based on the results of the STAIRWAY trial, which showed that nearly two-thirds of all faricimab-treated patients did not show disease activity 12 weeks after the last initiation dose and were eligible to try every-16-week dosing. In the STAIRWAY trial, a single disease activity assessment (based on OCT and BCVA criteria and investigator assessment) at week 24 enabled patients in the every-16-week arm with active disease to be dosed at an every-12-week interval.^30^ Introducing a second disease activity assessment time point in these phase 3 trials enabled patients with different treatment needs to be dosed at either every-8-week, every-12-week, or every-16-week intervals as required. After disease activity assessments at weeks 20 and 24 in the TENAYA and LUCERNE trials, patients randomized to the faricimab arm were assigned faricimab at fixed intervals of every 8 weeks, every 12 weeks, or every 16 weeks up to week 60.

The AVENUE trial demonstrated that faricimab 6.0 mg administered at every-8-week or every-4-week intervals was well tolerated and resulted in comparable vision and anatomic gains as those achieved with every-4-week ranibizumab. In a pharmacokinetic analysis of samples from the phase 2 trials, faricimab maintained high concentrations in the vitreous and demonstrated durable intraocular VEGF suppression in the aqueous humor for at least 8 weeks compared with 4 weeks with ranibizumab. In addition, sustained suppression of intraocular angiopoietin-2 was observed with faricimab. Although faricimab 6.0 mg every 4 weeks was shown to be well tolerated in the AVENUE trial, no efficacy advantage over every-8-week dosing was shown. For this reason, every-8-week dosing was selected as the minimum treatment interval in the TENAYA and LUCERNE trials based on disease activity status at week 20, and every 16 weeks was selected as the maximum treatment interval based on data from the STAIRWAY trial and pharmacokinetic and pharmacodynamic analyses from the phase 2 trials.^29^^,^^30^

### Faricimab Dosing Regimens During the Second Year of the Study: Objective and Rationale for the Personalized Treatment Interval Regimen

At week 60, all patients in the faricimab arm were scheduled to receive an active dose of faricimab and the second phase of the study began, in which faricimab-treated patients were dosed based on a PTI regimen. The PTI was a protocol-driven modified treat-and-extend (T&E) regimen with interval adjustment based on individualized treatment response as measured by CST and BCVA criteria. Treatment intervals could be extended in 4-week increments or reduced in 4- or 8-week increments to a minimum of every 8 weeks or a maximum of every 16 weeks or could be maintained according to calculations based on functional and anatomic criteria and clinical assessment by the investigator (Table 2; Fig S1) at study drug dosing visits. The PTI regimen was designed to tailor treatment intervals proactively according to patients’ disease activity and to reflect clinical practice, allowing more flexibility and personalization to reduce treatment burden while optimizing visual outcomes.

**Table 2:** Personalized Treatment Interval Phase Dosing Criteria

Dosing Interval	Criteria	Rationale for Decision
Interval extended by 4 wks (to a maximum of Q16W)	• Stable CST∗ compared with the average of the last 2 study drug dosing visits, and no increase ≥50 μm in CST (compared with lowest on-study drug dosing visit measurement) *and* • No decrease ≥5 letters in BCVA† compared with the average from the last 2 study drug dosing visits, and no decrease ≥10 letters in BCVA† compared with the highest on-study drug dosing visit measurement *and* • No new macular hemorrhage‡	Treatment interval increased when disease is stable
Interval reduced by 4 wks (to a minimum Q8W) If 1 criterion is met, the interval will be reduced by 4 wks. If >2 criteria are met or 1 criterion includes new macular hemorrhage, the interval will be reduced to an 8-wk interval§	• Increase ≥50 μm in CST compared with the average from the last 2 study drug dosing visits or ≥75 μm compared with the lowest on-study drug dosing visit measurement *or* • Decrease ≥5 letters in BCVA† compared with average of last 2 study drug dosing visits or decrease ≥10 letters in BCVA† compared with the highest on-study drug dosing visit measurement *or* • New macular hemorrhage‡	Treatment interval reduced in the event of disease reactivation (worsening of anatomic features, vision, or both)
Interval maintained	• If extension or reduction criteria have not been met	Treatment interval maintained if extension or reduction criteria not met

BCVA = best-corrected visual acuity; CST = central subfield thickness; Q8W = every 8 weeks; Q16W = every 16 weeks.

∗ Where stability is defined as a change of CST of <30 μm.

† Change in BCVA should be attributable to neovascular age-related macular degeneration disease activity (as determined by the investigator).

‡ Refers to macular hemorrhage owing to neovascular age-related macular degeneration activity (as determined by investigator).

§ Patients whose treatment interval is reduced by 8 weeks, from Q16W to Q8W, will not be allowed to return to a Q16W interval during the study.

**Figure 2:**
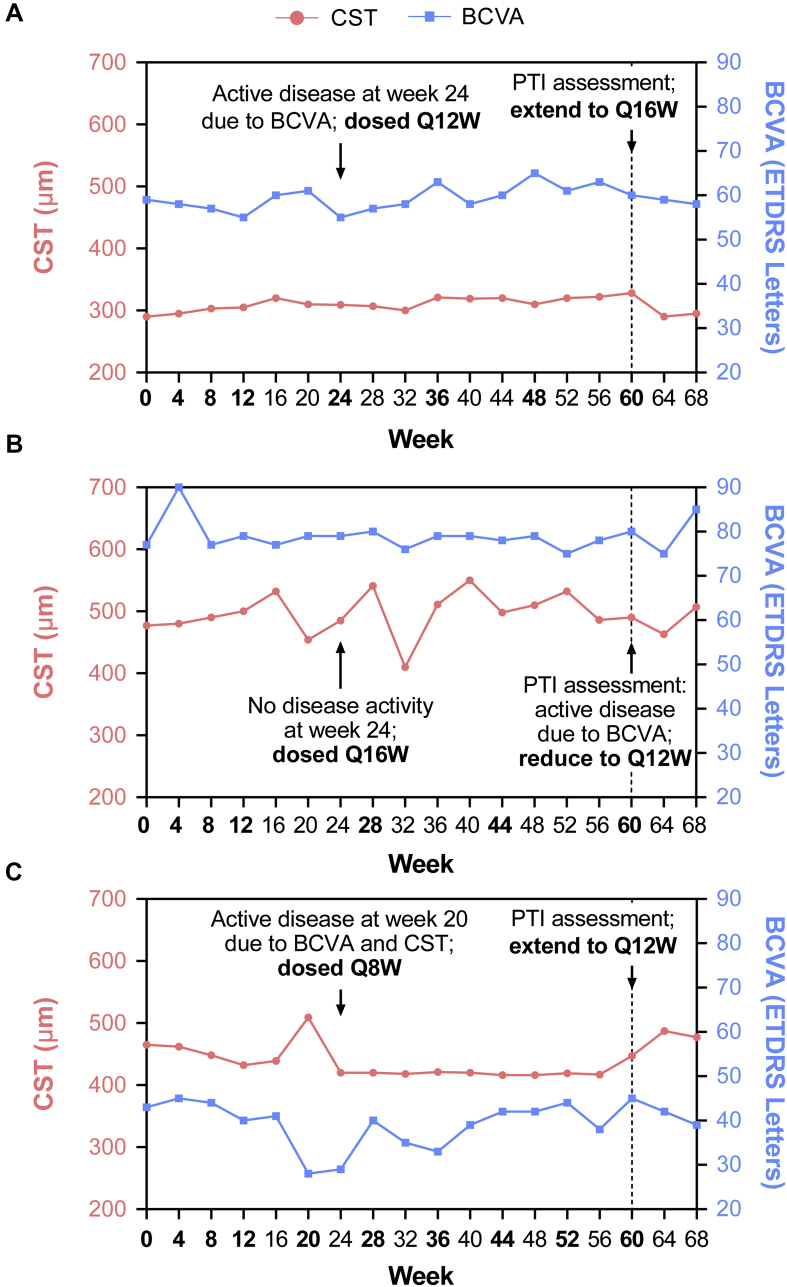
Personalized treatment interval (PTI) scenario examples. **A**, Disease activity resulting from best-corrected visual acuity (BCVA) met at week 24 and patient dosed every 12 weeks (Q12W) until week 60; at week 60, based on PTI assessment, patient meets the criteria for interval extension from Q12W to every 16 weeks (Q16W). **B**, No disease activity observed at weeks 20 and 24 and patient dosed Q16W until week 60; patient meets the PTI criteria for interval reduction at week 60, and interval reduced from Q16W to Q12W because of a 10-letter decrease in BCVA at week 60 compared with the highest on-study drug dosing measurement that is attributable to neovascular age-related macular degeneration disease activity. **C**, Active disease at week 20 resulting from both BCVA decrease and central subfield thickness (CST) increase compared with the previous 2 visits, resulting in the patient being dosed every 8 weeks (Q8W) until week 60; during the week 60 PTI assessment, patient meets the interval extension criteria from Q8W to Q12W. ETDRS = Early Treatment Diabetic Retinopathy Study.

For example, for a patient with an at least 10-letter decrease in BCVA, compared with the highest BCVA during a study drug dosing visit that was attributable to nAMD activity, the treatment interval was reduced by 4 weeks. In contrast, for a patient who showed a similar worsening in BCVA but also showed an at least 50-μm increase in CST compared with the average from the last 2 study drug dosing visits, the treatment interval was reduced by 8 weeks. Some scenario examples are illustrated in Figure 2.

### Study Outcomes and Rationale

The primary efficacy end point was the change from baseline in BCVA averaged over weeks 40, 44, and 48. The BCVA outcome measure is based on the Early Treatment Diabetic Retinopathy Study visual acuity chart assessed at a starting distance of 4 m. Averaging BCVA over 3 time points reduced the impact of measurement variability between visits, as well as intraobserver and interobserver variability. Thus, it may be a more robust measure of the true treatment effect on BCVA than measurement at a single time point, potentially providing a more precise measurement of treatment effect. Additionally, given the design of the TENAYA and LUCERNE trials, and therefore the variation in treatment schedules, averaging also minimized any potential impact of time since last active treatment on outcomes, and therefore allowed for a fairer comparison across treatment arms.

Secondary efficacy objectives included the proportion of patients receiving faricimab every 16 weeks, every 12 weeks, and every 8 weeks; evaluation of efficacy of faricimab on additional BCVA outcomes; and anatomic outcomes, such as change from baseline in CST over time, proportions of patients with absence of intraretinal fluid, and proportions of patients with absence of subretinal fluid over time. Other secondary objectives included the number of study drug injections received through weeks 48, 60, and 112. Preclinical evidence on dual angiopoietin-2 and VEGF-A inhibition suggests that the vessel-stabilizing effects of angiopoietin-2 impact vascular leakage and inflammation,^26^^,^^33^ which may reflect the efficacy of faricimab in these secondary outcome measures. Exploratory objectives focused on the evaluation of the efficacy of faricimab on patient-reported vision-related functioning and quality of life using the 25-item National Eye Institute Visual Functioning Questionnaire composite score outcome over time.

### Safety Assessments

One of the key objectives of the TENAYA and LUCERNE trials was to investigate the safety of faricimab. To ensure the safety of all patients during the conduct of the study, several safety assessments were included in the study design for year 1 and continue to be included through year 2. These include regular ophthalmologic assessment, adverse event monitoring, and protocol-specified laboratory safety tests. Additional safety imaging assessments were permitted at the principal investigator’s discretion, as required. An independent data monitoring committee monitored safety and study conduct on an ongoing basis.

All adverse events, including serious adverse events and adverse events of special interest, were required to be recorded on the adverse event electronic case report form and reported to the sponsor. Intraocular inflammation; infectious endophthalmitis associated with intravitreal injection; retinal detachment, tear, or both; iatrogenic traumatic cataracts; and increased intraocular pressure, as well as the potential nonocular risk of arterial thromboembolic events were adverse events of special interest based on experience with other intravitreal anti-VEGF monotherapies. Individual occurrences of these events were evaluated and documented by the study sites. The protocol defined the verbatim terms to be used when recording intraocular inflammation adverse events in the electronic case report form, which corresponded to Medical Dictionary for Regulatory Activities Preferred Terms. The Medical Dictionary for Regulatory Activities version 23.1 dictionary used in the study included the terms *ocular vasculitis* and *retinal vasculitis*. Cross-checks between the electronic case report form recorded clinical data and reports of adverse events were performed on an ongoing basis by the sponsor as part of the medical data review plan. Safety was assessed through descriptive summary of ocular and nonocular adverse events, deaths, and ocular assessments. Clinically significant laboratory abnormalities and clinically significant vital sign abnormalities were reported as adverse events and were evaluated as part of the adverse event assessments. Participant description of treatment-emergent adverse events was matched with Medical Dictionary for Regulatory Activities thesaurus terms, and the incidence and severity were summarized by treatment arm.

### Statistical Approaches

The study aimed to enroll a total sample size of approximately 320 patients per arm, which would provide more than 90% power to show noninferiority of faricimab compared with aflibercept in the intention-to-treat population, using a noninferiority margin of 4 letters in BCVA and under the following assumptions: no difference in the mean change from baseline in BCVA between the 2 treatment arms; standard deviation of 14 letters for the change from baseline in BCVA averaged over weeks 40, 44, and 48; 2-sample *t* test; 2.5% 1-sided type I error rate; and a 10% dropout rate. A nominal type I error penalty of 0.0001 was taken for each time the independent data monitoring committee reviews unmasked data before the formal analysis of the primary efficacy end point. The intention-to-treat population consisted of 1329 patients (TENAYA, n = 671; LUCERNE, n = 658) randomized in the study.

The per-protocol population consisted of all patients randomized who received at least 1 dose of study treatment and who did not have a major protocol violation that impacted the efficacy evaluation or treatment interval determination. The safety-evaluable population consisted of all patients who received at least 1 injection of active study drug (faricimab or aflibercept). Efficacy end points were analyzed using the intention-to-treat population, and safety outcomes were summarized using the safety-evaluable population. Additional efficacy analyses based on the per-protocol population also have been conducted.

Changes in BCVA from baseline (primary outcome) were compared using a mixed model for repeated measures, which assumed an unstructured covariance structure. The model included the change from baseline at weeks 4 to 48 as the response variable, the categorical covariates of treatment group, visit, visit by treatment group interaction, and baseline BCVA (continuous), as well as randomization stratification factors as fixed effects. Comparisons between the 2 treatment arms were made using a composite contrast over weeks 40, 44, and 48. Missing data were imputed implicitly by the mixed model for repeated measures. Although continuous secondary outcomes were analyzed using a mixed model for repeated measures, binary secondary end points were analyzed using stratified estimation for binomial proportions. The proportion of patients in each treatment group and the overall difference in proportions between treatment groups were estimated using the weighted average of the observed proportions and the differences in observed proportions over the strata defined by randomization stratification factor of baseline BCVA score, low-luminance deficit, and region. The estimates and confidence intervals were provided for the adjusted mean (for continuous variables) or weighted proportion (for binary variables) for each treatment group and for the difference between the 2 treatment groups. The efficacy analyses were tested at a significance level of 0.0497, and all confidence intervals were 2-sided and at the 95.03% level.

## Study Status

The TENAYA trial commenced recruitment in February 2019, and the LUCERNE trial commenced recruitment in March 2019. Primary end point analysis was completed for both trials in January 2021, and both studies are ongoing as of the date of this publication.

## Discussion

The TENAYA and LUCERNE trials are global phase 3 studies that enrolled more than 1300 patients with treatment-naïve nAMD to evaluate extended fixed treatment regimens of every-8-week to every-16-week faricimab immediately after the initial doses in the first year of the study based on disease activity assessment criteria. This description of the study design provides an understanding of the unique features of the TENAYA and LUCRENE trials, which were informed by the early clinical results for faricimab.

Although intravitreal anti-VEGF therapy targets the abnormal blood vessels and decreases permeability, potentially leading to regression of pathologic vasculature, it does not address inflammation and possibly fibrosis mediated by angiopoietin-2. Given the effects of angiopoietin-2 in vascular destabilization, neutralization of angiopoietin-2 combined with VEGF-A may have the potential to restore vascular stability and to induce maturation of vessels through restoring angiopoietin-1 and tyrosine kinase with immunoglobulin-like domains signaling, which in turn reduces vascular leakage, neovascularization, and inflammation, as well as vascular responsiveness to the effects of VEGF-A. Together, these effects contribute to a multitargeted approach to achieving vascular maturity compared with intravitreal anti-VEGF monotherapy.^24^ Evidence for sustained inhibition of vascular leakage and inflammation by anti–angiopoietin-2 and combined anti–angiopoietin-2 and anti–VEGF-A inhibition versus anti–VEGF-A alone from a mouse model of spontaneous CNV (JR5558 mice) supported the hypothesis that the sustained efficacy through extended durability demonstrated by faricimab in the phase 2 trials may be driven by its angiopoietin-2 inhibition properties,^26^^,^^33^ in addition to its VEGF-A blockade benefits. The dual specificity of faricimab may contribute to increased vascular stability and reduced inflammation. Less inflammation potentially may contribute further to reduced fibrosis as well as to reduced cell death, reducing subsequent atrophy.

In the phase 2 STAIRWAY trial, faricimab demonstrated sustained efficacy through extended durability on fixed every-12-week and every-16-week dosing, with comparable vision and anatomic gains versus every-4-week ranibizumab.^26^, ^27^, ^28^, ^29^, ^30^ The STAIRWAY trial highlighted the potential of faricimab to improve long-term outcomes in patients with nAMD, as demonstrated by the 65% of patients who did not show any disease activity at week 24, 12 weeks after the last initiation dose, suggesting that most patients potentially could be treated at an interval of at least every 12 weeks. It should be noted that patients with a higher upper BCVA cutoff of 78 to 24 letters (Snellen equivalent, 20/32–20/320) and a CNV lesion size of up to 9 disc areas on FFA were enrolled in the TENAYA and LUCERNE trials, as compared with 73 to 24 letters (Snellen equivalent, 20/40–20/320) and a CNV lesion size of up to 6 disc areas in the STAIRWAY trial to target a broader nAMD population. The upper limit of 78 letters in the TENYA and LUCERNE trials is in line with the general trend in current practice to treat patients earlier and not wait until they lose significant vision, as is evident in the change in recent clinical registrational trials such as HAWK and HARRIER, which included patients with a higher upper BCVA cutoff compared with older trials such as ANCHOR, MARINA, and VIEW.^2^, ^3^, ^4^^,^^7^

Alternate treatment regimens to fixed monthly dosing have been investigated in patients with nAMD, with a view to minimizing treatment burden and using a more individualized treatment approach.^5^^,^^21^^,^^34^, ^35^, ^36^, ^37^, ^38^ In the as-needed pro re nata regimen, monitoring intervals are fixed and frequent, and treatment decisions are based on anatomic and functional outcomes at each monitoring visit. However, visual outcomes with pro re nata dosing regimens have been shown to be significantly worse compared with those achieved with fixed monthly dosing after 2 years of treatment in the HARBOR trial and the Comparison of Age-related Macular Degeneration Treatments Trials.^13^^,^^39^ In T&E regimens, patients receive an injection at each visit, but the interval between follow-up visits is adjusted based on the disease activity at each visit, aiming to treat proactively before evidence emerges of further VEGF-driven disease activity.^40^ Several studies have demonstrated that visual outcomes achieved with T&E regimens were better than those achieved with pro re nata dosing, with results comparable with those achieved with monthly intravitreal anti-VEGF therapy and with fewer injections and, as such, fewer safety concerns.^34^^,^^38^^,^^41^, ^42^, ^43^, ^44^, ^45^ Furthermore, ranibizumab and aflibercept showed similar BCVA gains and numbers of injections during 1 year of a T&E regimen in patients with nAMD.^46^ The T&E dosing regimens have become widely used in clinical practice and are some of the most followed regimens worldwide.^47^ However, in clinical practice, standard T&E interval extensions generally are 2 to 4 weeks.^48^

In the TENAYA and LUCERNE trials, faricimab-treated patients follow a PTI approach from week 60. Extended treatment intervals adjusted based on patients’ individual responses to treatment allow personalized faricimab dosing, which may reduce the number of treatment visits, thereby reducing the treatment burden while still maximizing visual gains. This reflects a significant unmet need in patients with nAMD.^49^^,^^50^ Because the long-term benefits may not be fully apparent during the 112-week study period, an extension study (AVONELLE-X) is planned to allow follow up of patients completing the TENAYA and LUCERNE trials for a further 2 years. Additionally, faricimab is being studied for the treatment of diabetic macular edema and retinal vein occlusion in ongoing phase 3 clinical trials.

In conclusion, the phase 3 TENAYA and LUCERNE trials are evaluating the potential of faricimab, the first bispecific antibody that independently binds and neutralizes both VEGF-A and angiopoietin-2, to address an unmet clinical need for more durable therapies in nAMD that sustain BCVA gains over time.
