# Supplementary material for: TENAYA and LUCERNE: Rationale and Design for the Phase 3 Clinical Trials of Faricimab for Neovascular Age-Related Macular Degeneration
Source: Ophthalmol Sci. 2021 Nov 17;1(4):100076. doi: 10.1016/j.xops.2021.100076 (PMC9559073; doi:10.1016/j.xops.2021.100076)
Supplement: Fig S1 [file mmc1.pdf]

Supplementary information

eFigure S1. Personalized Treatment Interval Phase Dosing Criteria

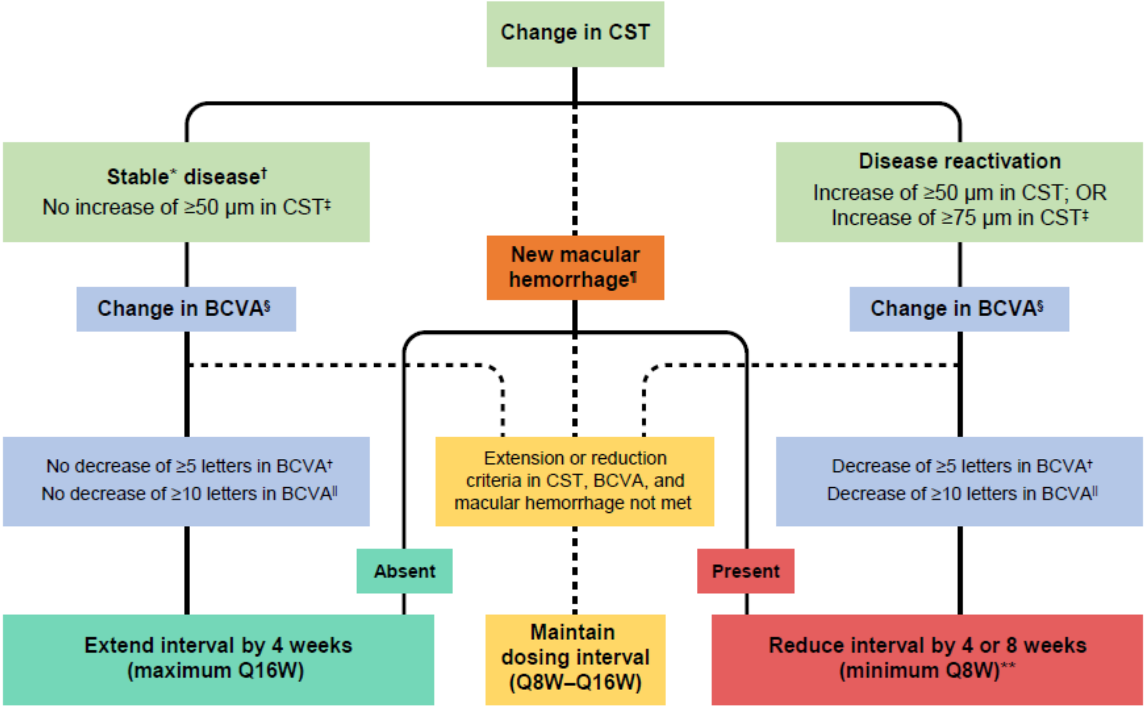

BCVA = best-corrected visual acuity; CST = central subfield thickness; Q8W = every 8 weeks; nAMD, neovascular age-related macular degeneration; Q16W = every 16 weeks.

*Where stability is defined as a change of CST of less than 30 µm.

†Compared with the average of the last 2 study drug dosing visits.

‡Compared with the lowest on-study drug dosing visit measurement.

§Change in BCVA should be attributable to nAMD disease activity (as determined by the investigator).

||Compared with the highest on-study drug dosing visit measurement.

¶Refers to macular hemorrhage owing to nAMD activity (as determined by investigator).

**Patients whose treatment interval is reduced by 8 weeks, from Q16W to Q8W, will not be allowed to return to a Q16W interval during the study.
