# Supplementary material for: TENAYA and LUCERNE: Rationale and Design for the Phase 3 Clinical Trials of Faricimab for Neovascular Age-Related Macular Degeneration
Source: Ophthalmol Sci. 2021 Nov 17;1(4):100076. doi: 10.1016/j.xops.2021.100076 (PMC9559073; doi:10.1016/j.xops.2021.100076)
Supplement: Table S1 [file mmc2.pdf]

Supplementary Information

eTable S1. Full List of institutional review boards (IRB)/ethics committees (EC) for TENAYA and LUCERNE trials

Investigator	IRB/EC Name and Address	Country
Karabas, Levent	Baskent University Clinical Research Ethics Committee	Turkey
Yilmaz, Gursel	Baskent University Clinical Research Ethics Committee	Turkey
Ozturk, Banu	Baskent University Clinical Research Ethics Committee	Turkey
Seres, András	Egeszsegugyi Tudomanyos Tanacs	Hungary
Herba, Ewa	Komisja Bioetyczna przy Slaskiej Izbie Lekarskiej w Katowicach	Poland
Muzyka-Wozniak, Maria	Komisja Bioetyczna przy Slaskiej Izbie Lekarskiej w Katowicach	Poland
Nowinska, Anna	Komisja Bioetyczna przy Slaskiej Izbie Lekarskiej w Katowicach	Poland
Wowra, Bogumil	Komisja Bioetyczna przy Slaskiej Izbie Lekarskiej w Katowicach	Poland
Varsanyi, Balazs	Egeszsegugyi Tudomanyos Tanacs	Hungary
Aradi, Etelka	Egeszsegugyi Tudomanyos Tanacs	Hungary
Oleksy, Piotr	Komisja Bioetyczna przy Slaskiej Izbie Lekarskiej w Katowicach	Poland
Becker, Matthias	Kantonale Ethikkommission Zürich (KEK), Kantonale Ethikkommission, Stampfenbachstrasse 121, 8090, Zürich, SWITZERLAND	Switzerland
Goldstein, Michaella	Helsinki Committee – Sourasky Tel Aviv; EC/IRB, 6 WEITZMAN STREET, 6423906, TEL AVIV, ISRAEL	Israel
Morori-Katz, Haia	Helsinki Committee - Kaplan	Israel
Patel, Praveen	Trent Multi-centre Research Ethics Committee	United Kingdom
Gale, Richard	Trent Multi-centre Research Ethics Committee	United Kingdom
Ghanchi, Faruque	Trent Multi-centre Research Ethics Committee	United Kingdom
Lotery, Andrew	Trent Multi-centre Research Ethics Committee	United Kingdom
Saedon, Habiba	Trent Multi-centre Research Ethics Committee	United Kingdom
Menon, Geeta	Trent Multi-centre Research Ethics Committee	United Kingdom
Mohamed, Quresh	Trent Multi-centre Research Ethics Committee	United Kingdom
Saeed, Usman	Trent Multi-centre Research Ethics Committee	United Kingdom
Cole, Abosede	Trent Multi-centre Research Ethics Committee	United Kingdom
Talks, James	Trent Multi-centre Research Ethics Committee	United Kingdom
Asaria, Riaz	Trent Multi-centre Research Ethics Committee	United Kingdom
Downey, Louise	Trent Multi-centre Research Ethics Committee	United Kingdom
Levy, Jaime	Helsinki Committee –Hadassah; EC/IRB	Israel
Agostini, Hansjurgen	Ethik-Kommission der Albert-Ludwigs-Universität, Engelberger Straße 21, 79106, Freiburg, GERMANY	Germany
Khoramnia, Ramin	EK Heidelberg, Alte Glockengießerei 11/1, 69115, Heidelberg, GERMANY	Germany

Völker, Michael	Ethik-Kommission der Medizinischen Fakultät und am, Gartenstraße 47, 72074, Tübingen, GERMANY	Germany
Ramirez Estudillo, Juan	CEI de Clinica Bajio CLINBA; Comite de Etica en Investigacion, Valenciana 7, Col. Paxtitlán. Guanajuato, Gto., 36090, Guanajuato, MEXICO	Mexico
Lozano Rechy, David	CEI de Clinica Bajio CLINBA; Comite de Etica en Investigacion, Calle Doctor Tomás Zavala No. 47, Col. Burócrata, Guanajuato, Gto., CP 36256, 36256, MARFIL, GUANAJUATO, MEXICO	Mexico
Montemayor Lobo, Rodrigo	CEI de Clinica Bajio CLINBA; Comite de Etica en Investigacion, Valenciana 7, Col. Paxtitlán. Guanajuato, Gto., 36090, Guanajuato, MEXICO	Mexico
Hatz, Katja	Ethikkommission Nordwest- und Zentralschweiz (EKNZ), Hebelstrasse 53, 4056, Basel, SWITZERLAND	Switzerland
Burgess, Stuart	Advarra, 6940 Columbia Gateway Drive, COLUMBIA, MD, 21046, UNITED STATES	United States of America
Huddleston, Stephen	Advarra, 6940 Columbia Gateway Drive, COLUMBIA, MD, 21046, UNITED STATES	United States of America
Campochiaro, Peter	Johns Hopkins Medicine Institutional Review Board, 1620 McElderry Street, Reed Hall B-130, Baltimore, MD, 21205-1911, UNITED STATES	United States of America
Carlson, John	Advarra, 6940 Columbia Gateway Drive, COLUMBIA, MD, 21046, UNITED STATES	United States of America
Chan, Clement	Advarra, 6940 Columbia Gateway Drive, COLUMBIA, MD, 21046, UNITED STATES	United States of America
Chang, Emmanuel	Advarra, 6940 Columbia Gateway Drive, COLUMBIA, MD, 21046, UNITED STATES	United States of America
Brown, Jamin	Advarra, 6940 Columbia Gateway Drive, COLUMBIA, MD, 21046, UNITED STATES	United States of America
Hershberger, Vrinda	Advarra, 6940 Columbia Gateway Drive, COLUMBIA, MD, 21046, UNITED STATES	United States of America
Higgins, Patrick	Advarra, 6940 Columbia Gateway Drive, COLUMBIA, MD, 21046, UNITED STATES	United States of America
Holekamp, Nancy	Advarra, 6940 Columbia Gateway Drive, COLUMBIA, MD, 21046, UNITED STATES	United States of America
Spencer, Doran	UCSD Human Research Protections Program; Altman Clinical and Translational Institute, 9452 Medical Center Drive, Level 2, LA JOLLA, CA, 92093, UNITED STATES	United States of America
Chaudhry, Nauman	Advarra, 6940 Columbia Gateway Drive, COLUMBIA, MD, 21046, UNITED STATES	United States of America
Cheek, Andrew	Advarra, 6940 Columbia Gateway Drive, COLUMBIA, MD, 21046, UNITED STATES	United States of America
Rich, Ryan	Advarra, 6940 Columbia Gateway Drive, COLUMBIA, MD, 21046, UNITED STATES	United States of America
Connolly, Brian	Advarra, 6940 Columbia Gateway Drive, COLUMBIA, MD, 21046, UNITED STATES	United States of America
Abbey, Ashkan	Advarra, 6940 Columbia Gateway Drive, COLUMBIA, MD, 21046, UNITED STATES	United States of America
Wong, David	St Mike's Hospital Research Ethics Board, 30 BOND STREET, M5B 1W8, TORONTO, Ontario, CANADA	Canada
Williams, Geoff	Health Research Ethics Board of Alberta, 1500, 10104-103 Ave NW, T5J 4A7, EDMONTON, Alberta, CANADA	Canada
Graff, Jordan	Advarra, 6940 Columbia Gateway Drive, COLUMBIA, MD, 21046, UNITED STATES	United States of America

Alfaro, Virgil	Advarra, 6940 Columbia Gateway Drive, COLUMBIA, MD, 21046, UNITED STATES	United States of America
Sheidow, Thomas	Western University Health Science Research Ethics Board	Canada
Danzig, Carl	Advarra, 6940 Columbia Gateway Drive, COLUMBIA, MD, 21046, UNITED STATES	United States of America
Dessouki, Amr	Advarra, 6940 Columbia Gateway Drive, COLUMBIA, MD, 21046, UNITED STATES	United States of America
Itty, Sujit	Advarra, 6940 Columbia Gateway Drive, COLUMBIA, MD, 21046, UNITED STATES	United States of America
Eichenbaum, David	Advarra, 6940 Columbia Gateway Drive, COLUMBIA, MD, 21046, UNITED STATES	United States of America
Engstrom, Robert	Advarra, 6940 Columbia Gateway Drive, COLUMBIA, MD, 21046, UNITED STATES	United States of America
Faber, David W.	Advarra, 6940 Columbia Gateway Drive, COLUMBIA, MD, 21046, UNITED STATES	United States of America
Falk, Naomi	Advarra, 6940 Columbia Gateway Drive, COLUMBIA, MD, 21046, UNITED STATES	United States of America
Dollin, Michael	Ottawa Ethics Board; Ottawa Health Science Network Research Ethics Board (OHSNREB), 725 Parkdale Avenue, Civic Box 675, K1Y 4E9, Ottawa, Ontario, CANADA	Canada
Feiner, Leonard	Advarra, 6940 Columbia Gateway Drive, COLUMBIA, MD, 21046, UNITED STATES	United States of America
Ferrone, Philip	Advarra, 6940 Columbia Gateway Drive, COLUMBIA, MD, 21046, UNITED STATES	United States of America
Shah, Sumit	Advarra, 6940 Columbia Gateway Drive, COLUMBIA, MD, 21046, UNITED STATES	United States of America
Fox, Gregory M.	Advarra, 6940 Columbia Gateway Drive, COLUMBIA, MD, 21046, UNITED STATES	United States of America
Chow, David	Advarra Inc., 300-372 Hollandview Trail, L4G 0A5, AURORA, Ontario, CANADA	Canada
Gasperini, Julie	Advarra, 6940 Columbia Gateway Drive, COLUMBIA, MD, 21046, UNITED STATES	United States of America
Gill, Manjot	Advarra, 6940 Columbia Gateway Drive, COLUMBIA, MD, 21046, UNITED STATES	United States of America
Glaser, David	Advarra, 6940 Columbia Gateway Drive, COLUMBIA, MD, 21046, UNITED STATES	United States of America
Gonzalez, Victor	Advarra, 6940 Columbia Gateway Drive, COLUMBIA, MD, 21046, UNITED STATES	United States of America
Gupta, Sunil	Advarra, 6940 Columbia Gateway Drive, COLUMBIA, MD, 21046, UNITED STATES	United States of America
Javid, Cameron	Advarra, 6940 Columbia Gateway Drive, COLUMBIA, MD, 21046, UNITED STATES	United States of America
Kelty, Patrick	Advarra, 6940 Columbia Gateway Drive, COLUMBIA, MD, 21046, UNITED STATES	United States of America
Almony, Arghavan	Advarra, 6940 Columbia Gateway Drive, COLUMBIA, MD, 21046, UNITED STATES	United States of America
Amini, Payam	Advarra, 6940 Columbia Gateway Drive, COLUMBIA, MD, 21046, UNITED STATES	United States of America
Antoszyk, Andrew	Advarra, 6940 Columbia Gateway Drive, COLUMBIA, MD, 21046, UNITED STATES	United States of America
Maberley, David	UBC Clinical Research Ethics Board, #210-828 West 10th Ave., V5Z 1L8, Vancouver, British Columbia, CANADA	Canada

Khanani, Arshad	Advarra, 6940 Columbia Gateway Drive, COLUMBIA, MD, 21046, UNITED STATES	United States of America
Cagini, Carlo	CER UMBRIA, Via Mario Angeloni, 61, Palazzo Broletto - 3 ° piano, 06124, Perugia, Umbria, ITALY	Italy
Viola, Francesco	Comitato Etico Milano Area 2, Via Francesco Sforza, 28, 20122, MILANO, Lombardia, ITALY	Italy
Lalonde, Laurent	Advarra Inc., 300-372 Hollandview Trail, L4G 0A5, AURORA, Ontario, CANADA	Canada
Parravano, Maria Cristina	CE Dell´IRCCS Istituti Fisioterapici Ospitalieri Di Roma, VIA ELIO CHIANESI 53, 00144, ROMA, Lazio, ITALY	Italy
Patel, Apurva	Advarra, 6940 Columbia Gateway Drive, COLUMBIA, MD, 21046, UNITED STATES	United States of America
London, Nikolas	Advarra, 6940 Columbia Gateway Drive, COLUMBIA, MD, 21046, UNITED STATES	United States of America
Awh, Carl C.	Advarra, 6940 Columbia Gateway Drive, COLUMBIA, MD, 21046, UNITED STATES	United States of America
Baumal, Caroline	IRB Tufts Medical Center; Tufts Univ Health Sciences, 800 Washington St., Box 450, boston, MA, 02111, UNITED STATES	United States of America
Reilly, Gayatri	Advarra, 6940 Columbia Gateway Drive, COLUMBIA, MD, 21046, UNITED STATES	United States of America
Bridges, Jr., William Z	Advarra, 6940 Columbia Gateway Drive, COLUMBIA, MD, 21046, UNITED STATES	United States of America
Lindsell, Lucas	Advarra, 6940 Columbia Gateway Drive, COLUMBIA, MD, 21046, UNITED STATES	United States of America
Brown, David M.	Advarra, 6940 Columbia Gateway Drive, COLUMBIA, MD, 21046, UNITED STATES	United States of America
Stoller, Glenn	Advarra, 6940 Columbia Gateway Drive, COLUMBIA, MD, 21046, UNITED STATES	United States of America
Yiu, Glenn	UC Davis IRB, 2921 Stockton Blvd., Suite 1429, Sacramento, CA, 95817, UNITED STATES	United States of America
Sikorski, Bartosz	Komisja Bioetyczna przy Slaskiej Izbie Lekarskiej w Katowicach	Poland
Vajas, Attila	Egeszsegugyi Tudomanyos Tanacs	Hungary
Banerjee, Sanjiv	Trent Multi-centre Research Ethics Committee	United Kingdom
Narendran, Niro	Trent Multi-centre Research Ethics Committee	United Kingdom
Budzinskaya, Maria	FSBI "Scientific Research Institute of Eye Diseases" of russia Academy of medical Sciences	Russia
Kulikov, Alexey	EC of FSB Military educational institution of HPE “Military Medical Academy n.a. S. M, Academic Lebedeva str., 6, 194044, Saint-Petersburg, RUSSIAN FEDERATION	Russia
Ito, Yasuo	Review Board of Human Rights and Ethics for Clinical Studies Institutional Review Board, 13-2 Ichibancho, Chiyoda-ku, 102-0082, Tokyo, JAPAN	Japan
Ito, Yasuki	Nagoya university Hospital IRB, 65 tsurumai-cho, showa-ku, nagoya-shi, 466-8560, Aichi, JAPAN	Japan
Iida, Tomohiro	Tokyo Women's Medical University Hospital IRB	Japan
Okada, Annabelle	Kyorin University Hospital Institutional Review Board, 6-20-2 Shinkawa, Mitaka, 181-8611, Tokyo, JAPAN	Japan
Hayashi, Ken	Hayashi Eye Hospital Institutional Review Board, 4-23-35 Hakata-eki mae, Hakata-ku, 812-0011, Fukuoka, JAPAN	Japan

Mitamura, Yoshinori	Tokushima University Hospital Institutional Review Board, 2-50-1 Kuramoto-cho, Tokushima-shi, 770-8503, Tokushima, JAPAN	Japan
Gomi, Fumi	Hyogo College of Medicine Institutional Review Board	Japan
Kobayashi, Namie	Southern TOHOKU General Hospital Institutional Review Board, 7-161 Yatsuyamada, Koriyama-shi, 963-8052, Fukushima, JAPAN	Japan
Ono, Shinji	Asahikawa Medical University Hospital Institutional Review Board, 1-1-1 Midorigaokahigashi2jo,Asahikawa-shi, 078-8510, Hokkaido, JAPAN	Japan
Wong, King	Stichting Beoordeling Ethiek Biomedisch Onderzoek (BEBO), Dr. Nassaulaan 10, 9401 HK, Assen, NETHERLANDS	Netherlands
van Lith-Verhoeven, Janneke	Stichting Beoordeling Ethiek Biomedisch Onderzoek (BEBO), Dr. Nassaulaan 10, 9401 HK, Assen, NETHERLANDS	Netherlands
Tuli, Raman	Advarra Inc., 300-372 Hollandview Trail, L4G 0A5, AURORA, Ontario, CANADA	Canada
Kawasaki, Tsutomu	Sugiura Clinic Institutional Review Board, 39 Nishitojinmachi Chuo-ku Kumamoto-shi, 860-0027, Kumamoto, JAPAN	Japan
Mori, Ryusaburo	Nihon University Hospital's Joint Institutional Review Board, 30-1 Oyaguchikamicho itabashi-ku, 173-8610, Tokyo, JAPAN	Japan
Maeno, Takatoshi	Toho University Sakura Medical Center Institutional Review Board, 564-1 Shimoshizu, Sakura-shi, 285-0841, Chiba, JAPAN	Japan
Oh, Hideyasu	Hyogo Prefectural Amagasaki General Medical Center (Hyogo AGMC) IRB, 2-17-77 Higashinaniwa-cho, amagasaki-shi, 660-8550, Hyogo, JAPAN	Japan
Yasuda, Kanako	Tokyo Medical University Hachioji Medical Center Institutional Review Board, 1163 Tatemachi, Hachioji-shi, 193-0998, Tokyo, JAPAN	Japan
Yoneda, Ai	Japanese Red Cross Nagasaki Genbaku Hospital Institutional Review Board, 3-15 Morimachi, Nagasaki-shi, 852-8511, Nagasaki, JAPAN	Japan
Kimura, Kazuhiro	Yamaguchi University Hospital Institutional Review Board, 1-1-1 Minami Kogushi Ube-Shi, 755-8505, Yamaguchi, JAPAN	Japan
Sekiryu, Tetsuju	All Tohoku Clinical Trial Review and Audit Organization, 1-6-10 kamisugi, aobaku, sendai-shi, 980-0011, Miyagi, JAPAN	Japan
Sakamoto, Taiji	Kagoshima University Hospital Institutional Review Board, 8-35-1 Sakuragaoka, Kagoshima-shi, 890-8520, Kagoshima, JAPAN	Japan
Giunta, Michel	Advarra Inc., 300-372 Hollandview Trail, L4G 0A5, AURORA, Ontario, CANADA	Canada
Honda, Shigeru	Osaka City University Hospital IRB, 1-5-7,Asahimachi, Abeno-ku, Osaka-shi, 545-8586, Osaka, JAPAN	Japan
Abraham, Prema	Advarra, 6940 Columbia Gateway Drive, COLUMBIA, MD, 21046, UNITED STATES	United States of America
Takahashi, Kanji	Kansai Medical University Hospital Institutional Review Board, 2-3-1 Shinmachi, Hirakata-shi, 573-1191, Osaka, JAPAN	Japan
Margherio, Alan	Advarra, 6940 Columbia Gateway Drive, COLUMBIA, MD, 21046, UNITED STATES	United States of America

Mehta, Sonia	Wills Eye Hospital IRB, 840 Walnut St., 15th Floor, Philadelphia, PA, 19107, UNITED STATES	United States of America
Takayama, Kei	National Defense Medical College Hospital Institutional Review Board, 3-2 Namiki, Tokorozawa-shi, 359-8513, Saitama, JAPAN	Japan
Matsubara, Hisashi	Mie University Hospital Institutional Review Board	Japan
Garcia-Layana, Alfredo	CEIC HU Puerta de Hierro de majadahonda, Planta 1ª - Pasillo Unidades Administrativas (peines 6-7), 28222, Majadahonda Madrid, MADRID, SPAIN	Spain
Ruiz Moreno, Jose Maria	CEIC HU Puerta de Hierro de majadahonda, Planta 1ª - Pasillo Unidades Administrativas (peines 6-7), 28222, Majadahonda Madrid, MADRID, SPAIN	Spain
Escobar, Joan Josep	CEIC HU Puerta de Hierro de majadahonda, Planta 1ª - Pasillo Unidades Administrativas (peines 6-7), 28222, Majadahonda Madrid, MADRID, SPAIN	Spain
Gomez Ulla, Francisco	CEIC HU Puerta de Hierro de majadahonda, Planta 1ª - Pasillo Unidades Administrativas (peines 6-7), 28222, Majadahonda Madrid, MADRID, SPAIN	Spain
Dolz, Rosa	CEIC HU Puerta de Hierro de majadahonda, Planta 1ª - Pasillo Unidades Administrativas (peines 6-7), 28222, Majadahonda Madrid, MADRID, SPAIN	Spain
Figueroa, Marta	CEIC HU Puerta de Hierro de majadahonda, Planta 1ª - Pasillo Unidades Administrativas (peines 6-7), 28222, Majadahonda Madrid, MADRID, SPAIN	Spain
Sararols, Laura	CEIC HU Puerta de Hierro de majadahonda, Planta 1ª - Pasillo Unidades Administrativas (peines 6-7), 28222, Majadahonda Madrid, MADRID, SPAIN	Spain
Murata, Toshinori	Shinshu University Hospital Institutional Review Board, 3-1-1 Asahi, Matsumoto-shi, 390-8621, Nagano, JAPAN	Japan
Abdulaeva, Elmira	LEC of Ltd."Kuzlyar", Chistopolskaya str., 16/15, 420066, KAZAN, TATARSTAN, RUSSIAN FEDERATION	Russia
Nishimura, Tetsuya	Kansai Medical University Hospital Institutional Review Board	Japan
Noda, Kousuke	Hokkaido University Hospital Institutional Review Board, Kita14-jo,Nishi5-chome,Kita-ku,Sapporo, 060-8648, Hokkaido, JAPAN	Japan
Koizumi, Hideki	University of the Ryukyus Hospital Institutional Review Board	Japan
Yamashita, Ayana	Kagawa University Hospital Institutional Review Board, 1750-1, Ikenobe, Miki-cho, Kita-gun, 761-0793, Kagawa, JAPAN	Japan
Garcia-Layana, Alfredo	CEIC HU Puerta de Hierro de majadahonda, Planta 1ª - Pasillo Unidades Administrativas (peines 6-7), 28222, Majadahonda Madrid, MADRID, SPAIN	Spain
Kaga, Tatsushi	Japan Community Health care Organization Chukyo Hospital, 1-1-10, Sanjo Minami-ku, Nagoya-shi, 457-8510, Aichi, JAPAN	Japan
Imaizumi, Hiroko	Sapporo City General Hospital Institutional Review Board, 3-32-8 Shinjuku, Shinjuku-ku, 060-8604, Hokkaido, JAPAN	Japan
Yoreh, Barak	Helsinki Committee - Rambam	Israel
Rosenblatt, Irit	Helsinki Committee - Rabin Beilinson M.C	Israel
Mukherjee, Raj	Trent Multi-centre Research Ethics Committee	United Kingdom
Gawecki, Maciej	Komisja Bioetyczna przy Slaskiej Izbie Lekarskiej w Katowicach	Poland

EC = ethics committee; IRB = institutional review board.
