# Supplementary material for: TENAYA and LUCERNE: Rationale and Design for the Phase 3 Clinical Trials of Faricimab for Neovascular Age-Related Macular Degeneration
Source: Ophthalmol Sci. 2021 Nov 17;1(4):100076. doi: 10.1016/j.xops.2021.100076 (PMC9559073; doi:10.1016/j.xops.2021.100076)
Supplement: Table S2 [file mmc3.pdf]

**Supplementary Information**

eTable S2. Full List of Inclusion and Exclusion Criteria

| **Inclusion Criteria** | |
| --- | --- |
| Ocular criteria for study eye | • Treatment-naïve, active CNV secondary to AMD (nAMD)<br>• Subfoveal CNV or juxtafoveal/extrafoveal CNV with a subfoveal component related to the CNV activity by FFA or SD-OCT<br>• CNV lesion of all types (predominantly classic, minimally classic, or occult [including polypoidal choroidal vasculopathy and retinal angiomatous proliferation]) with:<br>  o Total lesion size (including blood, atrophy, fibrosis, and neovascularization) of ≤9 disc areas by FFA; and<br>  o CNV component area of ≥50% of total lesion size by FFA; and<br>  o Active CNV confirmed by FFA (evidence of leakage); and<br>  o CNV exudation confirmed by SD-OCT (presence of fluid)<br>• BCVA letter score of 78–24 letters (inclusive) on ETDRS-like charts (20/32–20/320 Snellen equivalent) using the ETDRS protocol and assessed at the initial testing distance of 4 m on day 1<br>• Clear ocular media and adequate pupillary dilatation to allow acquisition of good quality retinal images to confirm diagnosis |
| General criteria | • Signed informed consent form<br>• Age ≥50 years on day 1 (screening)<br>• Ability to comply with the study protocol, in the investigator's judgment |

| | |
|---|---|
| | • For women of childbearing potential: agreement to remain abstinent (refrain from heterosexual intercourse) or use a contraceptive method with a failure rate of <1% per year during the treatment period and for ≥28 days after the last dose of study treatment<br>o A woman was considered to be of childbearing potential if she was postmenarcheal, had not reached a postmenopausal state (≥12 continuous months of amenorrhea with no identified cause other than menopause), and had not undergone surgical sterilization (removal of ovaries and/or uterus)<br>o Examples of contraceptive methods with a failure rate of <1% per year include bilateral tubal ligation, male sterilization, hormonal contraceptives that inhibit ovulation, hormone-releasing intrauterine devices, and copper intrauterine devices<br>o The reliability of sexual abstinence was evaluated in relation to the duration of the clinical trial and the preferred and usual lifestyle of the patient. Periodic abstinence (e.g., calendar, ovulation, symptothermal, or postovulation methods) and withdrawal were not acceptable methods of contraception<br>• Current residents of mainland China, Hong Kong, or Taiwan for patients enrolled in the China extension |
| **Exclusion Criteria** | |

| | |
|---|---|
| Ocular criteria for study eye | • CNV due to causes other than AMD, such as ocular histoplasmosis, trauma, pathological myopia, angioid streaks, choroidal rupture, or uveitis<br>• Any history of macular pathology unrelated to AMD affecting vision or contributing to the presence of intraretinal or subretinal fluid<br>• Central serous chorioretinopathy at screening<br>• Retinal pigment epithelial tear involving the macula on day 1<br>• On FFA/CFP:<br>  o Subretinal hemorrhage of >50% of the total lesion area and/or that involved the fovea<br>  o Fibrosis or atrophy of >50% of the total lesion area and/or that involved the fovea<br>• Any concurrent intraocular condition (amblyopia, aphakia, retinal detachment, cataract, diabetic retinopathy or maculopathy, or epiretinal membrane with traction) that, in the opinion of the investigator, could either reduce the potential for visual improvement or require medical or surgical intervention during the study<br>• Current vitreous hemorrhage on day 1<br>• Uncontrolled glaucoma<br>• Spherical equivalent of refractive error demonstrating >8 diopters of myopia<br>• Any previous or concomitant treatment for CNV or vitreomacular-interface abnormalities, including (but not restricted to) IVT treatment (steroids, anti-VEGF, tissue plasminogen activator, ocriplasmin, $C_3F_8$ gas, air), periocular pharmacological |

| | |
|---|---|
| | intervention, argon laser photocoagulation, verteporfin photodynamic therapy, diode laser, transpupillary thermotherapy, or surgical intervention<br>• Cataract surgery or treatment for complications of cataract surgery with steroids or YAG laser capsulotomy within 3 months of baseline assessments (day 1)<br>• Any other intraocular surgery (pars plana vitrectomy, glaucoma surgery, corneal transplant, radiotherapy)<br>• Previous periocular pharmacological or IVT (including anti-VEGF medication) treatment for other retinal diseases |
| Exclusion criteria for both eyes | • Previous IVT administration of faricimab in either eye<br>• History of idiopathic or autoimmune-associated uveitis in either eye<br>• Active ocular inflammation or suspected or active ocular or periocular infection in either eye on day 1 |
| General criteria | • Any major illness or major surgical procedure within 1 month before screening<br>• Active cancer within the 12 months before day 1 except for appropriately treated carcinoma in situ of the cervix, non-melanoma skin carcinoma, and prostate cancer with a Gleason score of ≤ 6 and a stable prostate-specific antigen for > 12 months<br>• Requirement for continuous use of any prohibited medications and treatments<br>• Systemic treatment for suspected or active systemic infection on day 1 (ongoing use of prophylactic antibiotic therapy may be acceptable if approved after discussion with the Medical Monitor) |

- Uncontrolled blood pressure (defined as systolic >180 mmHg and/or diastolic >100 mmHg while the patient is at rest). If a patient's initial reading exceeded these values, a second reading was taken later on the same day, or on another day during the screening period. If the patient's blood pressure was controlled by antihypertensive medication, the patient was taking the same medication continuously for ≥30 days before day 1
- Stroke or myocardial infarction within 6 months before day 1
- History of other disease, metabolic dysfunction, physical examination finding, or clinical laboratory findings giving reasonable suspicion of a condition that contraindicated the use of the investigational drug or that might affect interpretation of the results of the study or renders the patient at high risk for treatment complications in the opinion of the investigator
- Pregnant or breastfeeding, or intended to become pregnant during the study
    - Women of childbearing potential had to have a negative urine pregnancy test result within 28 days before initiation of study treatment. If the urine pregnancy test was positive, it was confirmed by a serum pregnancy test
- Known hypersensitivity to any component of the faricimab or aflibercept injections, study-related procedure preparations (including fluorescein), dilating eye drops, or any of the anesthetic and antimicrobial drops used
- History of a severe allergic reaction or anaphylactic reaction to a biologic agent or known hypersensitivity to any component of the faricimab or aflibercept injections, study-related procedure

| | |
|---|---|
| | preparations (including fluorescein), dilating drops, or any of the anesthetic and antimicrobial preparations used by a patient during the study<br>• Participation in an investigational trial that involved treatment with any drug or device (except with vitamins and minerals) within 3 months before day 1 |

AMD = age-related macular degeneration; BCVA = best-corrected visual acuity; CFP = color fundus photography; CNV = choroidal neovascularization; ETDRS = Early Treatment Diabetic Retinopathy Study; FFA = fundus fluorescein angiography; IVT = intravitreal; nAMD = neovascular age-related macular degeneration; SD-OCT = spectral domain OCT; VEGF = vascular endothelial growth factor; YAG = yttrium-aluminum-garnet.
